# From obese to lean curriculum: exploring students’ experiences about developing competencies in medical education

**DOI:** 10.3389/fmed.2024.1309548

**Published:** 2024-05-22

**Authors:** Morteza Karami, Nooriyah Hashemi, Jeroen Van Merrienboer

**Affiliations:** ^1^Department of Curriculum Studies and Instruction, Ferdowsi University of Mashhad, Mashhad, Iran; ^2^English Department, Herat University, Herat, Afghanistan; ^3^Department of Educational Development and Research, Maastricht University, Maastricht, Netherlands

**Keywords:** CBME, undergraduate medical curricula, curriculum change, medical curricula, competency development

## Abstract

**Introduction:**

Since the beginning of the 21st century, competency-based education has been proposed as an approach to education in many disciplines including the medical sciences and it has become a dominant approach in many countries. We aimed to explore the lived experiences of general medical students about developing competencies in the academic curriculum.

**Methods:**

We conducted a phenomenology method to study lived experiences of general medical students through selecting participants via a purposeful sampling strategy. Snowballing and maximum variation samplings were also applied to recruit additional participants. The study was conducted at a Medical School in Iran. Three successive phases of qualitative data analysis, namely, data reduction by coding, data structuring by categorization, and data interpretation by discussion were applied to analyze the interviews.

**Results:**

The results of the research showed that students’ lived experiences fall under 4 main themes with 9 subthemes. The main themes show that (1) the compartmentalized curriculum in basic courses is experienced as the missing parts in a puzzle, (2) the physiopathology curriculum is experienced as swimming on land, (3) the externship is experienced as touring a mysterious land, (4) the internship is experienced as unleashed arrows.

**Discussion:**

Our findings reveal that despite the changes already made in the curriculum, its compartmentalization is still a main obstacle to achieving competency-based medical education. A strict requirement for leaving the discipline-based curriculum behind is to use an integrated approach, in which basic science courses are connected with clinical cases, and physiopathology courses are connected with externships and internships.

## Introduction

Health professions education can be considered important because these jobs play an essential role in the well-being of individuals, families, and communities (1). Rapid developments in the area of medical and health services, advances in information technology, globalization, and changing political and public expectations have caused medical education to face more pressures for change than ever before (2). These pressures have had clear implications for curriculum change and revision (3), one of the most important of which has been the adoption of an integrated and competency-based approach in the development and revision of medical curricula (4, 5).

Since the beginning of the 21st century, competency-based medical education (CBME) has become a dominant approach to medical education in many countries (6–8). Globally there is a move from a structure and process-based approach to CBME at all levels of the medical training system (9, 10). One reason for the widespread acceptance of this approach is its merits (11). Competency-based curricula offer structural, content, and process advantages. Benefits include a focus on learners’ outcomes and progress, formative and observation-based assessment, support for flexible learning, and an increase in transparency and responsiveness to all stakeholders with a set of shared expectations and a common language for learning (12). However, many universities still offer a discipline-based curriculum that is similar to the curriculum that Flexner considered more than a hundred years ago. For them, the realization of a competency-based curriculum is not an easy task and has all the features of complex change (13). Accordingly, it is necessary to examine the challenges of transitioning from a discipline-based to a competency-based curriculum.

In health care settings, curricula should be able to develop professional competencies such as those listed in CanMEDS (14), the Accreditation Council of Graduate Medical Education from the US (15) or Good medical practice (16). It appears that in discipline-based learning environments - still practiced in many medical schools and designed to teach theoretical classroom content without considering practical work in the real world (17) – there are many challenges in this regard. Focusing on academic disciplines, segmentation of theoretical knowledge and practical skills, and emphasis on learning the logic or basic structure of each major - key concepts, principles, and relationships - are among the main features of a discipline-based curriculum (18).

CBME has important implications for re-organizing the medical curriculum (19). Competency development is a form of complex learning because it involves the integration of knowledge, skills, and attitudes in individuals so that they can demonstrate effective performance in clinical practice. Complex learning requires the coordination of qualitatively different skills and the transfer of what is learned into daily life or the workplace (20). Learners develop professional competencies when working on meaningful learning assignments. Competency development relies on experience and coping with real-world tasks (21). Task-centered learning environments provide a good alternative to learning professional competencies in clinical practice. In such environments, learners focus on working on learning tasks that are based on professional tasks in both the classroom (e.g., using role play and simulation) and the workplace (22). By combining on-the-job learning with off-the-job learning, it integrates in-school with out-of-school learning opportunities and thus better prepares learners for their future profession (23, 24).

In this task-centered model, ‘dual blended learning’ aims to achieve an optimal combination of instructional methods by distinguishing two levels of blends: The first level combines face-to-face training with online training and the second level combines simulation-based training with workplace or real-world training (20). In such environments, education and assessment are aligned with each other, meaning that the learning tasks that learners focus on are not just for training but also a vehicle for feedback and assessment. The basic idea of this approach is that such tasks help learners to integrate knowledge, skills, and attitudes, motivate them to learn constituent skills, and facilitate the transfer of what they have learned to new situations (25, 26).

One of the main features of a competency-based curriculum is that students are involved in their learning process, that is, in how their competencies develop over tasks and over time, rather than focusing on the demarcated acquisition of discipline-based subject matter (27). Accordingly, reviewing students’ experiences of the implemented curriculum can provide clear guidelines for revising and improving the curriculum.

The study of CBME issues has generated increasing attention and debate among researchers and practitioners in the health professions in recent years (19), but few studies examined the problems of developing competencies from a students’ perspective. Therefore, the purpose of this study is to explore the lived experience of general medical students about the learning environment in which they study. The results of this study will provide insightful guidelines for improving the quality of the medical curriculum.

## Methods

### Research design overview

We used a phenomenology research method for the study. This qualitative study was undertaken using in-depth semi-structured interviews with medical students at Mashhad University of Medical Sciences, Mashhad, Iran. Mashhad University of Medical Sciences is one of the largest and most advanced universities in Iran, organized in 25 clinical and 19 basic science departments. All fifth- and sixth-year medical students who were passing externship (clerkship) and internship courses were invited to participate in the study.

In 2015, Iran’s Ministry of Health and Medical Education (MHME) announced that the institutions of higher education must ensure all graduates of the medical programs can demonstrate professional commitment, decision-making, and problem-solving (clinical skills), as well as communication skills, sensitivity to caring for patients, self-regulation skills for individual development or continuous learning, and the ability to improve community health. To meet the new educational aims, a new curriculum was developed by introducing the core competencies. One of the important criticisms of the old curriculum of medical education in Iran was that it was more concentrated on biological, clinical, and natural sciences. Thus, it lacked an emphasis on integrated and applied knowledge and skills, as well as the social, emotional, and ethical components of becoming a physician. In the revised curriculum, the emphasis is on the integration of the basic and clinical courses in order to prepare students for professional responsibilities by using a variety of teaching methods including problem-based learning, task-based learning, outpatient-based education, and community-oriented education. Also, in the new curriculum, some courses are defined for key skills including communication skills, critical thinking, team working. In addition, despite the centralized curriculum system in Iran, the new curriculum allows medical schools to develop optional courses.

Researchers’ characteristics were important in the design of our study. The lead author (First author), who prompted the study design and did the data collection, as a curriculum specialist, made a contribution to the curriculum development at the institutional level. Our research team also had a medical education leader (Third author) who conceptualized the study, and a research coordinator (Second author) who contributed her significant educational research experience through coordinating data analysis.

### Participants

To identify and select the participants, a purposeful sampling strategy was used. Snowballing and maximum variation samplings were also applied to recruit additional participants. At the end of each interview, the participants were invited to suggest other potential participants who might be willing to share their unique lived experiences with us. Table 1 describes the participants’ characteristics. Moreover, no relationship was established between the interviewer and the participants prior to the study.

**Table 1:** The participants’ characteristics.

Participant	Characteristics
A	Female, 23 years, an externship student
B	Female, 23 years, an externship student
C	Male, 26 years, an externship student
D	Female, 27 years, an externship student
E	Female, 25 years, an externship student
F	Male, 24 years, an externship student
G	Female, 28 years, an externship student
H	Female, 24 years, an externship student
I	Male, 25 years, an externship student
J	Female, 29 years, an externship student
K	Male, 23 years, an externship student
L	Female, 26 years, an externship student
M	Male, 24 years, an externship student
N	Male, 27 years, an externship student

### Data collection

Given the goals of the current research and the curriculum characteristics (i.e., after the intended transition toward a competency-based curriculum), the interview guide was developed. The interviews began with the request, “Tell me about your experience in medical school.” Then we asked students to share their lived experience in spending basic sciences, physiopathology, internships, and internships.

The students who expressed their willingness to participate were informed about the interviews and the researchers’ reasons and interests for this study. They were, then, asked to attend the individual interviews after signing the informed consent forms. All interviews were conducted by the first author who is a professional expert of curriculum studies.

All interviews were conducted in the department of medical education at the Mashhad University of Medical Sciences. Face-to-face interviews were used to gather information and, during the interviews, no one except the participants and the researcher was present. All invited students participated (100%). Each interview lasted approximately 30–45 min. Notes were taken by the researcher and all interviews were audio-recorded. After each individual interview, the content was checked to find out if new information emerged. The interviews were then transcribed word by word. The transcripts were returned to the participants for comments and corrections and the interviewing process continued up to the point of saturation. All data were saved confidentially and were only accessible to the investigators. This study was carried out in Iran in accordance with the applicable rules concerning the review of research ethics committees and informed consent. The consent forms were written and signed by all the participants before conducting the interviews. The signed forms were confidentially archived.

### Analysis

Three successive phases of qualitative data analysis based on Miles and Huberman’s theory (28) were applied to analyze the interviews. These phases include data reduction by coding, data structuring by categorization, and data interpretation by discussion, respectively. All interview transcripts were transferred into the MAXQDA software package and all items were coded by second author. The codes were utilized as the first coding dictionary. First author revised the coding dictionary by removing the code duplicates and discussing the codes. First author organized the codes and discussed their organization with third author to identify the different aspects of the learning goals, teaching, and assessment. During the analysis process, sub-themes were created and/or reduced by merging them. This allowed the analysis to reach internal homogeneity and external heterogeneity. The questioning and challenging of the emerging themes continued in an iterative process via the thematic analytical model by going back and forth between the researchers’ assumptions, ideas, questions, and explanations and, finally, a validation of these themes through comparing them with the transcripts. The analysis was continuously discussed and re-evaluated by the authors (All authors) to improve the reliability of the analysis through the examination of different dimensions, contradictory information, and interpretations. The participants were not asked to provide feedback on the findings. The data interpretation through discussion was the linking activity throughout the whole analysis process and during the decision-making process about the pertinent quotes.

## Results

In this section, we will describe the students’ experiences about the medical curriculum. We identified four themes and nine subthemes as described in Table 2.

**Table 2:** The themes and subthemes.

Theme	Subtheme
Compartmentalized curriculum in basic courses is like the missing parts in a puzzle	Unrelated subjects of basic courses to medical workplace
	Theoretical-based classes
Physiopathology curriculum is like swimming in the land	Gaps between physiopathology and externship lessons
	Concentrating on exam and assessment more than learning
Externship as touring to mysterious land	A broken bridge between experiences of class and workplace
	Inappropriate authenticity
Internship and unleashed arrows	Myopic view
	Chaotic learning environment in hospitals
	Core competency like clinical reasoning, problem solving, and making decisions as null curriculum

### Compartmentalized curriculum in the basic courses is like the missing parts in a puzzle

The experience of students in the first years of studying in medical school is simply attending classes to take basic courses such as biochemistry, physiology, anatomy, and so on. From the students’ point of view, these courses seem unnecessary because they have no specific connection with the clinic. As an example, student B stated:

“The basic science program was such that many of its courses, such as biochemistry and medical physics, were not very important and could have been presented more succinctly, and in my opinion, nothing would have happened if these courses had not been offered. The courses are taught by many professors mainly in the form of theory.”

Student F similarly stated:

“Basic science courses, anyone we ask says, are not important courses; they are useless because they have a series of lessons that have very little to do with the clinic in terms of content, and those parts that are related to the clinic, students do not understand the connection.”

Students believe that the large volume of content in the basic courses and holding theoretical classes in crowded classrooms yielded an undesirable form of education and this, in turn, has reduced their motivation. Student E stated:

“In the basic science course, we were not at a desirable level in terms of class population. For example, physiology class, which was also an important subject, was held with 120 students in the amphitheater. Who really wanted to listen to the class or take notes or whatever? Classes were like this, and so was the lab. For example, we had the anatomy classes like the anatomy of the body or the head and neck, in practice, but the classes were so crowded, and on the other hand, the number of corpses were so limited that it was not possible to work much on the corpses or to know precisely what was going on. The other classes were also the same.”

Student H mentioned:

“In total, the basic science course was not useful. Apart from this aspect of theoretical knowledge, it did not motivate or encourage questioning in any way, and it was mostly because the volume of the lessons was large and the things we had to read were too much. The volume of lessons was so overwhelming that we preferred to read only the pamphlets and books we had and we no longer had time for further why and how questions.”

What can be concluded from the students’ experiences is that the compartmentalization of the curriculum has led to the implementation of basic courses with a purely theoretical approach, with no connection to clinical practice. Accordingly, these courses are offered in crowded classrooms with passive teaching methods.

#### Physiopathology curriculum is like swimming on land

Although students believe that recognizing diseases is the basis of medical science, they believe that the current way of instruction is like swimming on land. They mentioned gaps between physiopathology and externship courses and concentrating on exams and assessments rather than learning as the main reasons for the current situation.

The participants identified a need for selecting appropriate interventions for closing the gaps between physiopathology and externship lessons. They explained that their courses in physiopathology courses are not helpful for their externship period. Student K explained:

“I think physiopathology and externship lessons are not aligned with the needs of students. It would be better that physiopathology lessons to be adjusted with an externship. It should be mentioned that clinical subjects are also theory-based.”

Student G also described her attitude as:

“The physiopathology course is an intensive and difficult course in which a lot of things are told to students in a short time, so the most fundamental thing that students do is to memorize their booklets, take the exam, and pass. Professors do not guide us how to link our knowledge to clinical skills to have effective outcomes to use in our workplace.”

The participants explained that the learning environment is designed in a way that professors focus on exams and assessments more than learning. Student C stated:

“Memorizing chapters more than concentrating on learning confuses the students. Students spend their time and energy for just taking their exams and remembering the heavy content to pass the courses.”

The participants also described that concentrating on theory-based classes is a chance for the students to cheat on the exam questions from senior students. They also described those theory-based classes as the cause of decreasing students’ motivation. Student N made this point:

*“Experiencing theoretical courses for 5 years decreases students’ motivation. Students* do not understand the reasons for memorizing a great amount of details *and it is the main cause of decreasing students’ motivation.”*

The findings of this section show that despite the importance of physiopathology courses in the diagnosis and treatment of diseases as the main medical function, the isolation from the action arena makes this part of the curriculum ineffective.

#### Externships as touring to mysterious land

After 4 years in the classroom taking basic and physiopathology classes, going to the hospital and wearing a white medical uniform is very exciting for students. They attend in different wards of the hospital, waiting for opportunities to turn them into doctors, but what they experience is like a trip to Wonderland. They expect the knowledge they have gained over the years will act as a bridge helping them to transfer from university to the real therapeutic (medical) environment; however, they feel that the bridge is broken. For example, student G stated:

“We expected all the different courses we took in college to prepare us for real-life issues in the workplace, but our experience does not show that at all.”

They believe that the learning environment is very specialized and far from the expectations of a general practitioner, and this has led to inappropriate authenticity of learning assignments. For example, student J stated:

“We go to one of the most specialized hospitals in the city to do an internship. Patients who are referred to this hospital have advanced and specific diseases while I am supposed to be a general practitioner and treat common diseases that do not exist in such hospitals.”

Student A stated:

“There are various learning opportunities in the hospital, such as Morning Report, Professor Case, and Clinical Round, but since all of these situations are the subject of advanced and specialized disease, they are not applicable to our future work. On the other hand, in all these situations, in addition to general medicine students, there are also residents and fellowships, and these situations are more relevant to them than to us and the supervisor pays more attention to them.”

Students believe that there is a deep gap between what is learned in the classroom and practice in clinical settings. In addition, attending various wards of a specialized hospital prevents them from learning the duties of a general practitioner, and what is offered is more appropriate for a resident than for a future general practitioner.

#### Internships and unleashed arrows

Re-attendance of students in different treatment departments in specialized hospitals as interns is an opportunity to learn and perform therapeutic interventions. However, students believe that attending specialized departments has caused them to see diseases only from the specialized perspective of that department, and this has led to the formation of a myopic view in them. For example, student C stated:

“In every ward we work, they only analyze the disease from their point of view. For example, if the patient refers to the ear, nose, and throat ward with symptoms such as headache and nausea, the disease is examined only from the perspective of the same organs of the body, and if he refers to the internal ward, the symptoms are analyzed only from the gastrointestinal dimension while I, as a future general practitioner, should have a holistic view of the disease.”

Students also state that due to a large number of patients in the ward and improper management of time and tasks, proper planning is not done for their internship, and learning in such an environment depends on their luck and effort. According to this, student D stated:

“We spend many hours in the hospital every day, but there is no specific schedule for us. If I show enthusiasm and I am lucky and the resident who is my supervisor gives me a chance to perform the treatment procedures, then, I will have a good learning opportunity. Otherwise, time often passes in vain.”

In this regard, student I stated:

“There are good topics for learning in the curriculum, but due to a large number of patients, professors, and residents are engaged in treatment, and learning depends on the extent that I am an active observer.”

Students are aware that core competencies such as clinical reasoning and problem-solving should be developed by them and they have been emphasized in the curriculum. However, they believe that, in practice, there is no opportunity to learn such skills during their internship. Core competencies are not considered at all so a unique opportunity for their development is missed. For example, student L stated:

“We know that we need skills such as reasoning, problem-solving, and decision-making to make the right diagnoses, or that professional ethics are important, but in practice, these issues are not considered by supervisors during the study, neither in teaching nor in assessment.”

Students expect to practice the duties of a general practitioner in a related work environment during their internship period. However, attending only specialized hospitals and the lack of proper supervision by professors has turned the learning environment into a chaotic one where learning is more influenced by chance and luck than by being influenced by the program and the teacher. Moreover, in internships, specialization takes precedence over the development of core competencies such as clinical reasoning and problem-solving.

## Discussion

This study focused on analyzing the lived experiences of general medical students about a curriculum that is in a process of change, from discipline-based to competency-based. The findings of this study are significant and show that the experiences of our participants reflect the challenges of designing a learning environment that helps students develop medical competencies. Understanding the unique environment of clinical education and how it affects learning and performance brings greater clarity to the students’ lived experiences of the medical curriculum. These findings and the inclusion of trainees in this research can drive conversations on future curriculum change. The remainder of the takeaways are interesting and beneficial for the field.

The students’ lived experiences of taking basic courses show that they consider these courses as being unnecessary because, in their perception, they have nothing to do with the clinic. This experience can be a natural result of a discipline-oriented curriculum that focuses more on academic disciplines than anything else (18). We introduce the concepts of the *obese curriculum* and *appended curriculum* to describe these experiences of students in basic courses because, on the one hand, the curriculum is full of courses that have no clear relationship with medical competencies and, on the other hand, in this type of curriculum, the quantity of knowledge taught in each lesson is emphasized. This, in turn, makes much of the content, despite being informative in its own right, not clearly related to the real world of the medical profession. Developing professional competencies requires a *lean curriculum*; a curriculum that is outcome-oriented and the mission of each course in the curriculum is to help students develop specific and core competencies. At the level of courses, the role of each learning activity, students’ assignments, and assessment methods should be aligned with the expected outcomes of the course, which is to educate and assess competencies.

In addition, students stated that taking basic science courses in the classroom without being associated with the clinical setting reduced their motivation. One of the disadvantages of the disciplinary approach is the separation of the curriculum into pre-clinical and clinical sections, which makes the student in the first academic years have no experience of being in the environment and seeing real patients, and this deeply frustrates students (17) (29). In addition, the results of the research show that a lack of integration of anatomy courses with the clinic prevents near and far transfer of learning (30). All physicians need knowledge of the basic sciences, although this level of need varies between different specialties. Balancing clinical and basic sciences and, especially, fully integrating them in a way that best serves the competency development of medical students is an issue that needs to be the focus of many future innovations (31, 32).

For physiopathology courses, we introduce the concept of the *isolated curriculum* because the curriculum prevents learning to diagnose and treat diseases in connection with the real clinical environment. The main reason for this issue seems to be the ‘vertical organization’ of the curriculum. One of the main goals of vertical integration is to facilitate the transfer of classroom learning to clinical training (33). In the conventional form of vertical organization of a medical curriculum, based on a disciplinary approach, scientific theories, and basic knowledge are taught in the pre-clinical years and clinical years, and clinical skills are taught. This type of organization represents the traditional H-shaped medical curriculum (34). However, a more effective form of vertical integration should include the presentation of early clinical experiences, the integration of biomedical sciences and clinical cases, the gradual increase of clinical responsibility, and the increase of internships in the final year of study (33). An innovative alternative including these features is the Z-shaped curriculum model in which, in the first 2 years, the basic sciences are taught in combination with clinical cases. In the third year, more attention is paid to practical and clinical skills and elective courses. Regular internships are scheduled in the fourth and fifth years. Research shows the positive effects of this type of curriculum on learning (35). The Z-shaped curriculum is also in line with 4C/ID model (20), with many simulation-based learning tasks (role play, virtual patients, and standardized patients) and relatively few real-life learning tasks in the early years, but many real-life learning tasks (internships) and relatively few simulation-based learning tasks in the later years.

What students’ experiences show is that they have to adapt to the requirements of the curriculum throughout their studies, and this is one of the features of the much-criticized disciplinary curriculum (18). In contrast, a well-designed competency-based curriculum should put the students and their process of competency development in the center, rather than the subject matter taught (27).

Students stated that competencies such as clinical reasoning and problem-solving were neglected in the curriculum. Studies highlight the difficulty of translating a competency framework into concrete, integrated curricula for students (36). One of the biggest drawbacks of the disciplinary approach is that it causes educational systems to ignore the vast amounts of learning goals that cannot be classified as ‘regular knowledge’. Of course, it should be emphasized that the well-meant purpose of the disciplinary design approach is to nurture students who can use acquired knowledge to produce understanding and thoughtful action. However, students’ lived experiences indicate that they feel this approach emphasizes the “filling” of their minds rather than the development of clinical reasoning and problem-solving skills.

Students asserted that, during their internships, they were not able to apply in the real world what they had learned in the foregoing years. This experience can be seen as a direct result of compartmentalization and fragmentation in the curriculum, which seriously hampers the transfer of learning (37). A chaotic learning environment, inappropriate authenticity of learning assignments, and the formation of a myopic view were other harms of internships from the student’s point of view. Competency development should be explicitly embedded into the curriculum and cannot be left to individual instructors (36). Applying a task-centered approach to the design of learning environments can be a good alternative to escape from this situation and to realize a *lean curriculum* because it offers a structured approach to making real-world problems the driving force of learning (38). The purpose of task-centered instruction is to apply and transfer knowledge to the real context and to learn effectively and efficiently (23). In a task-centered learning environment, the link between classroom learning and workplace learning can be well established (20). Daniel et al. (39) and Vandewaetere et al. (21) describe successful examples of this approach in medical education.

Contrary to the disciplinary approach in which individual professors or faculty curriculum committees are typically responsible for their courses and curriculum revisions (40), the compilation and revision of a task-oriented curriculum in medical education is teamwork that requires the effective participation of curriculum planners and users, especially professors and students (21). Because students’ learning is driven by tasks that are based on real-life authentic tasks, multidisciplinary teacher teams must possess all knowledge necessary for students to - learn to - perform these tasks. This, in turn, can pave the way for the effective implementation of these programs.

To our knowledge, this is one of the few qualitative studies into transition challenges from a discipline-based to a competency-based curriculum by analyzing student experiences. This study also provides insights into the requirements of revising the medical curriculum (e.g., transitioning from an H-shaped to a Z-shaped curriculum by applying principles from task-centered instruction). Yet, it was limited to the education of general practitioners in Iran, where, despite moving toward a competency-based curriculum, the disciplinary approach still plays an essential role. Although this type of curriculum is common in many universities, it cannot represent the general medical curriculum worldwide. Though our results are derived from a local context, we think it could have clear implications for improving the quality of medical learning environments.

## Conclusion

Compartmentalization is the main obstacle to achieving a competency-based curriculum. The main requirement for leaving a discipline-based curriculum behind is to use an integrated approach in which basic science courses are connected with clinical cases, and physiopathology courses are connected with externships and internships. To link the development of specialized and core competencies in the curriculum, adopting a task-centered learning approach is appropriate. This approach is also fully compatible with the Z-shaped model of the medical curriculum because it emphasizes the increasing authenticity of learning assignments during the study period.

## Data availability statement

The original contributions presented in the study are included in the article/supplementary material, further inquiries can be directed to the corresponding author.

## Ethics statement

The studies involving humans were approved by the Mashhad University of Medical Sciences University. The studies were conducted in accordance with the local legislation and institutional requirements. The participants provided their written informed consent to participate in this study.

## Author contributions

MK: Data curation, Methodology, Project administration, Supervision, Writing – review & editing. NH: Formal analysis, Software, Writing – original draft. JM: Conceptualization, Methodology, Validation, Writing – review & editing.
